# The synergy of morphokinetic parameters and sHLA-G in cleavage embryo enhancing implantation rates

**DOI:** 10.3389/fcell.2024.1417375

**Published:** 2024-07-16

**Authors:** Chunyan Jiang, Menghui Geng, Can Zhang, Hong She, Dalin Wang, Jing Wang, Jiayin Liu, Feiyang Diao, Lingbo Cai, Yanqiu Hu

**Affiliations:** ^1^ State Key Laboratory of Reproductive Medicine and Offspring Health, Clinical Center of Reproductive Medicine, The First Affiliated Hospital of Nanjing Medical University, Nanjing, China; ^2^ Reproductive Medicine Center of Northern Jiangsu People’s Hospital, Yangzhou, China; ^3^ Department of Obstetrics and Gynecology, The 2^nd^ Affiliated Hospital and Yuying Chidren’s Hospital of Wenzhou Medical University, Wenzhou, China; ^4^ Department of Obstetrics and Gynecology, Tumor Hospital Affiliated to Nantong University, Nantong, China; ^5^ Reproductive Medicine Center of Xu Zhou Maternity and Child Healthcare Hospital Affiliated to Xuzhou Medical University, Xuzhou, China

**Keywords:** soluble human leukocyte antigen G (sHLA-G), time-lapse imaging (TLI), embryo implantation, embryo selection, embryo grade

## Abstract

**Objective:** This study aimed to assess the relationship between implantation and soluble HLA-G (sHLA-G) expression in cleavage embryo culture medium (ECM) in conjunction with early developmental kinetics determined by time-lapse imaging (TLI).

**Methods:** A retrospective, single-center study was conducted involving 238 embryos from 165 patients who underwent Frozen-thawed embryo transfer (FET) using autologous oocytes, with either single or double embryo transfer. TLI morphokinetic parameters (t2, t3, t4, t5, t6, t7, t8, cc2, s2, cc3, s3) of embryos were analyzed, and sHLA-G levels in D3 ECM were measured using an enzyme-linked immunosorbent assay (ELISA). A hierarchical classification model was developed to categorize embryos into five groups (A, B, C, D, E). The correlation between sHLA-G levels, TLI classification of embryos, and embryo implantation was investigated to establish a non-invasive method for evaluating implantation potential. Multivariate logistic regression analysis was performed to identify potential influencing factors, and receiver operating characteristic (ROC) curves were used to evaluate the predictive value for implantation.

**Results:** Multivariate unconditional logistic regression analysis indicated that TLI parameters t5 and s3 and sHLA-G level in ECM were independent risk factors affecting embryo implantation. The implantation rate decreased from TLI classification A to E. The proposed classification model effectively assessed the implantation potential of embryos. The implantation rate was higher in the sHLA-G positive group compared to the sHLA-G negative group (*p* < 0.001). The expression of sHLA-G in D3 ECM, combined with the TLI classification model, accurately evaluated the implantation potential of embryos with an AUC of 0.876.

**Conclusion:** The integration of cleavage kinetics and embryonic sHLA-G expression could reliably identify embryos with a high likelihood of successful implantation.

## Introduction

*In vitro* fertilization-embryo transfer (IVF-ET) technology has emerged as a primary auxiliary technique for infertility treatment. Despite advancements in treatment methodologies and laboratory technologies, the pregnancy rate for patients undergoing their first IVF-ET treatment can reach up to 60%. However, some patients still experience failure after multiple IVF-ET treatments, a phenomenon closely associated with repeated implantation failure (RIF) (ESHRE Working Group on Recurrent Implantation Failure et al., 2023). Poor quality embryos/blastocysts and morphokinetic abnormalities are linked to decreased reproductive competence, even in the context of euploid ETs (Shear et al., 2020; Bamford et al., 2022). Yet, due to the absence of large-scale random studies and concrete treatment effects, systematic treatment measures are still lacking (ESHRE Working Group on Recurrent Implantation Failure et al., 2023). Embryo quality is a crucial determinant of pregnancy outcome, and the selection of embryos with the highest developmental potential for transfer poses a significant challenge for embryologists. Numerous studies have indicated a high incidence of fetal aneuploidy in RIF patients, making preimplantation screening (PGS) a logical treatment choice for this population (Cozzolino et al., 2020; Tong et al., 2021). However, due to chromosomal mosaicism and the invasive nature of biopsies, robust evidence supporting the routine use of PGS in RIF patients is lacking. In the “ESHRE good practice recommendations on recurrent implantation failure,” Preimplantation genetic testing for aneuploidies (PGT-A) is deemed as a consideration. However, embryo grading is limited and largely determined by static morphologic criteria (Cimadomo et al., 2022; Fordham et al., 2022).

To overcome the limitation of embryo scoring system, non-invasive evaluation methods, including embryo viability molecular-biomarkers, dynamic time-lapse evaluation, Raman spectroscopy assess, are being considered (Meng et al., 2023). Non-invasive evaluation methods do not disrupt the normal developmental environment of the embryo, nor do they negatively impact or directly damage the embryo. Recently, time-lapse systems (TLS), which capture digital images of embryos at regular intervals, have been developed (Kovacs, 2014; Gallego et al., 2019; Sciorio and Meseguer, 2021). These systems enable embryologists, with or without the aid of embryo selection software, to assess embryo quality without physically removing them from the incubator. Consequently, TLS serves as an ideal tool to study the dynamic biological processes of embryo development. It facilitates the selection of embryos at more advanced developmental stages and provides morphological, dynamic, and quantitative timing data, i.e., morphokinetic parameters (Goodman et al., 2016; Curchoe and Bormann, 2019). With the broad application of TLS in Assisted Reproductive Technology (ART), embryologists are dedicated to establishing the relationship between morphokinetic parameters and embryonic developmental potential and pregnancy outcomes (Liu et al., 2015; Bamford et al., 2022).

Soluble human leukocyte Antigen G (sHLA-G) is a non-classical HLA-like molecule that is specifically highly expressed on the trophoblastic layer in the early human placenta. Although the role of HLA-G in the preimplantation embryo remains ambiguous, it has been proposed to be involved in immunoprotection, adhesion, and cell signaling mechanisms (Shaikly et al., 2010). Fuzzi et al. found that sHLA-G secreted by embryos could be a developmental potential marker, with its concentration varying depending on the ovarian stimulation protocol used (Fuzzi et al., 2002). Other researches also indicated that sHLA-G has an impact on implantation and live birth (Noci et al., 2005). Utilizing the Graduated Embryo Score and soluble human leukocyte Antigen G (sHLA-G) for Day 3 embryo transfer enhances Assisted Reproductive Technology (ART) outcomes by augmenting predictive accuracy (Fisch et al., 2007). sHLA-G status to traditional morphological criteria may be useful as a clinical tool for embryo selection.

Despite the higher pregnancy rate associated with blastocyst transfer, there is currently no evidence to suggest that cleavage-stage embryos unable to form blastocysts cannot lead to successful pregnancies. Additionally, studies have indicated that blastocyst transfer may lead to a reduction in telomere length in offspring (Wang et al., 2022). Therefore, cleavage-stage embryo transfer remains widely employed in clinical practice.

In the current study, we introduced a clinical investigation involving time-lapse system (TLS) morphokinetic parameters of embryo development and the measurement of soluble human leukocyte Antigen G (sHLA-G) protein levels in Day 3 (D3) embryo culture medium for 238 transferred embryos. This research proposes an innovative non-invasive approach that combines time-lapse parameters with sHLA-G in the cleavage medium of embryos to ascertain their implantation potential.

## Materials and methods

### Study design

This study was conducted on a cohort of infertile patients who underwent Frozen Embryo Transfer (FET) at the Reproductive Medicine Center of Northern Jiangsu People’s Hospital between May 2017 and September 2018. The inclusion criteria encompassed women aged 20–44 years who had at least one embryo available for transfer. Furthermore, only cycles demonstrating either 100% implantation success or 100% implantation failure were considered. Exclusion criteria consisted of congenital or acquired uterine anomalies, endometrial polyps and submucosal fibroids, intrauterine adhesion, severe endometriosis or adenomyosis, systemic diseases such as diabetes mellitus, and endocrine disorders such as hyperprolactinemia. Adhering to these criteria, 146 couples were enrolled in the study. The study protocol received approval from the ethics committee of Northern Jiangsu People’s Hospital. Informed consent was obtained from all participating patients.

### Embryo culture and time-lapse recording

Fertilization was assessed 16–18 h after insemination. All normally fertilized zygotes (2PN) were placed individually into a CCM-iBIS (12-Well miniGPS^®^, Japan) at 37°C, 6.0% CO_2_ and 5.0% O_2_ and were cultured to D3. All Zygotes were cultured separately using sequential media (G-1 Plus, Vitrolife; Goteborg, Sweden) in 25 μL microdrops under oil. The precise timing of numerous morphokinetic parameters was identified. Since the study included IVF and ICSI cycles, it was difficult to quantify the time from insemination, so we considered the time of pronuclear fading (tPNF) as the time of initiation (Liu et al., 2015). The absolute times of division to 2, 3, 4, 5, 6, 7 and 8 cells minus the tPNF were defined as t2, t3, t4, t5, t6, t7, and t8, respectively. We also calculated the cell cycle duration, including cc2, the length of the 2-cell period, cleavage from 2- to 3-cell; s2, the duration of cell division from 3- to 4-cell; cc3, time of the third cell cycle, cleavage from 3- to 5-cell; and s3, the development of 5-cell embryos into 8-cell embryos. In particular, if we observed direct or rapid cleavage from one to 3 cell (i.e., CC2<5 h), direct uneven cleavage (DUC), irregular chaotic division (ICD), or reverse cleavage (RC) phenomena, those embryos were defined as having irregular division patterns.

### Embryo grade

Embryos were evaluated and selected based on cell morphology (CM) selection on Day 3 (D3) using the following grading system: Grade 1, embryos with blastomeres of equal size and less than 5% fragmentation; Grade 2, embryos with blastomeres of equal or unequal size and less than 20% fragmentation; Grade 3, embryos with blastomeres of unequal size and 20%–50% fragmentation; and Grade 4, embryos with few blastomeres of any size and more than 50% fragmentation. Embryos of Grade 1–3, which contained at least 6 cells on D3, were deemed suitable for use. Following a comprehensive evaluation of patient characteristics, experienced clinicians determined whether patients underwent fresh embryo transfer or a freeze-all procedure.

### Embryos vitrification and thawing procedures

The vitrification procedures were executed in accordance with the manufacturer’s instructions (KITAZATO^®^ Vitrification Kit, Japan) at ambient temperature (22°C–28°C). Embryos were immersed in the equilibrium solution (ES, KITAZATO^®^ Vitrification Kit Code.VT102-01) for a duration of 5 min, followed by the vitrification solution (VS, KITAZATO^®^ Vitrification Kit Code.VT102-02). Upon thorough mixing, the embryos were loaded onto the apex of the Cryotop (KITAZATO^®^ Cryotop package) and subsequently submerged in liquid nitrogen.

The embryo thawing procedures were conducted on a heated platform maintained at 37°C. Straws were extracted from the liquid nitrogen and immersed in the thawing solution (TS, KITAZATO^®^ Vitrification Kit Code.VT102-01) for 1 min, followed by the diluent solution (DS, KITAZATO^®^ Vitrification Kit Code.VT102-02) for 3 min. Subsequently, they were placed in washing solution 1 (WS1, KITAZATO^®^ Vitrification Kit Code.VT102-03) for 5 min and finally in washing solution 2 (WS2, KITAZATO^®^ Vitrification Kit Code.VT102-04) for 5 min. Ultimately, embryos were transferred to a fresh feeder layer containing microdroplets of cleavage medium (G-1 Plus, Vitrolife; Goteborg, Sweden).

### Embryo transfer

Post-thawing, the quality of the embryos was re-evaluated. Survival was characterized by the preservation of at least 50% of intact cells. Embryos with a minimum of 6 cells and less than 20% intracellular fragmentation were classified as high quality. Suitable embryos were cultured for a duration of 2 h, after which they were transferred into the uterine cavity under ultrasound guidance, with 1–2 embryos per procedure.

### Pregnancy assessment

All patients underwent daily progesterone administration following Frozen Embryo Transfer (FET). Early pregnancy confirmation was achieved by measuring serum hCG levels 14 days post-embryo transfer, and conducting ultrasonography between days 28 and 35 post-FET. Implantation and Clinical Pregnancy (CP) were defined by the presence of a gestational sac, with or without a detectable fetal heartbeat. A miscarriage was characterized as the loss of a CP, while a Live Birth (LB) was defined as the delivery of a viable infant post 28 weeks of gestation.

### Sample collection and sHLA-G measurement

Post-embryo removal on Day 3 (D3), the 20 μL culture media were promptly collected into a 0.5 mL Eppendorf tube and cryopreserved at −80°C. A total of 238 embryo culture media (ECM) samples were gathered. A control medium, incubated under identical conditions but without any embryo, was also collected for comparison. To determine embryonic sHLA-G levels, an Enzyme-Linked Immunosorbent Assay (ELISA) technique (BioVendor GmbH, Heidelberg, Germany) was employed according to manufacturer’s instructions. The antibody for sHLA-G was mouse monoclonal anti-human sHLA-G antibody MEM-G/9.

### Statistical analysis

The continuous data were analyzed for normality with Shapiro-Wilk test. Univariate analysis was used with Student’s t-test when the data were normally distributed; otherwise, a nonparametric test (Mann-Whitney U-test) was used. Descriptive statistics were expressed as the means, standard deviations, percentage distributions, medians, and interquartile ranges. Chi-square test was used to compare the rates. The significant parameters of univariate analysis will be taken into a logistic regression analysis. The odds ratio (OR) of the effect of the variables generated on implantation was expressed in terms of a 95% confidence interval (95% CI) and significance. To define the ECM samples as sHLA-G-positive and to determine the optimal times ranges of t5 and s3, the cut-off values were determined regarding the optimal values of sensitivity and specificity for the prediction of implantation using receiver operating characteristic (ROC) curve analysis. The results were expressed as ROC using AUC values to establish the predictive strength of the model presented here. AUC values between 0.5 and 1 and can be interpreted as a measurement of the global classification ability of the model. A further analysis with an independent data was performed to verify the TLI classification model. All statistical calculations were carried out using the Statistical Package for the Social Sciences software SPSS 20.0 (IBM Inc., New York, Armonk): *p* < 0.05 was considered significant.

## Results

### Patients’ characteristics

This study incorporated data from 165 couples that underwent IVF/ICSI-FET. No significant disparities were observed between the implantation group (*n* = 82) and the non-implantation group (*n* = 83) with respect to the following clinical characteristics: age, Body Mass Index (BMI), infertility duration, types of infertility, fertilization method, basal serum Follicle Stimulating Hormone (FSH), Luteinizing Hormone (LH), Estradiol (E2) levels, antral follicles, endometrial thickness, duration of gonadotropin use, total dosage of gonadotropins and the number of transferred embryos. Yet, as delineated in Table 1, the implantation group exhibited a markedly superior number of retrieved oocytes and those at the Metaphase II (MII) stage in comparison to the control group.

**TABLE 1:** Baseline clinical characteristics for all patients.

Characteristics	Patients in Implantion Group (n=82)	Patients in Non-Implantion Group (n=83)	*p*-value
Maternal age (years)	28 (27, 30.25)	29 (27, 32)	0.348
Maternal BMI (Kg/m^2^)	22.18 ± 3 .27	22.76 ± 2.70	0.220
Infertility duration (years)	3 (2, 4)	3 (2, 3)	0.277
Types of infertility			
Primary, n (%)	46 (56.10)	50 (60.24)	0.637
Secondary, n (%)	36 (43.90)	33 (39.76)	
bFSH (IU/L)	5.99 (5.04, 6.66)	5.47 (4.69, 6.63)	0.216
bE_2_ (IU/L)	128.85 (91.77, 169.73)	133.60 (106.50, 165.10)	0.330
bLH (IU/L)	5.42 (4.17, 6.28)	4.42 (3.60, 5.85)	0.058
No. of antral follicle (n)	11 (8, 15)	10 (8, 13)	0.088
Days for gonadotropin (days)	9 (8, 10)	9 (7, 10)	0.711
Total gonadotropin dose (IU)	2138.41 ± 525.10	2165.06 ± 601.46	0.762
Endometrium thickness (mm)	11 (9, 12)	11 (9.6, 12.0)	0.885
No. of embryo transferred			
One, n (%)	53 (64.63)	39 (46.99)	0.231
Two, n (%)	29 (35.37)	44 (53.01)	
No. of oocytes retrieved (n)	11 (7, 12.25)	8 (6, 12)	0.009^*^
No. of MII oocytes (n)	8.5 (6, 13.25)	8 (5, 10)	0.024^*^
Fertilization			
IVF, n (%)	65 (79.27)	70 (84.34)	0.426
ICSI, n (%)	17 (20.73)	13 (15.66)	

Note: BMI, body mass index; bFSH, basal Follicle Stimulating Hormone; bE2, basal estradiol; bLH, basal Luteinizing hormone; IVF, In vitro fertilization; ICSI, Intracytoplasmic sperm injection. Data are presented as the mean ± SD or number (%),or median (quartiles). The (*) values indicate the significant differences between the two groups (*p* < 0.05).

### Morphokinetic parameters analysis

To reduce inconsistencies in early cleavage times, we employed the time of disappearance of the pronuclei (tPNF) as the initial reference and compared the morphokinetic parameters of implanted and non-implanted embryos to analyze the potential application of these parameters in predicting embryo implantation ability.

Embryo abnormal cleavage events: Among the 238 embryos that were transplanted, 25 of them experienced early abnormal cleavage, including direct cleavage: 1 cell dividing into 3 cells, cc2 < 5 h; reverse cleavage: two blastomeres refusing after completion of division or incomplete blastomeres eventually fusing. Among them, only 3 embryos successfully implanted, but no live births were observed. Therefore, embryo abnormal cleavage can be used as an exclusion criterion for embryo selection.

Various time parameters between Implantation and Non-Implantation Embryos: t2, t4, t6, t7, and t8 show no significant difference between the two groups. The morphokinetic end points, specifically t5, were attained earlier, whereas t3 was reached later in the implantation group. Additionally, we computed the duration of the cell cycle and discovered that s2 and cc3 were shorter, in contrast to the extended durations of CC2 and S3 in the implantation group compared to the non-implantation group. (refer to Table 2 for more details).

**TABLE 2:** Comparison of morphokinetic parameters (hours) between implantation and non-implantation group.

	Implantation group *n* = 109	Non-implantation group *n* = 129	*p*-value
t2	2.38 ± 0.88	2.36 ± 0.48	0.871
t3	12.75 ± 2.28	11.61 ± 3.57	0.004^*^
t4	14.22 ± 1.78	13.98 ± 2.86	0.448
t5	20.75 (18.75, 25.50)	26.25 (24.00, 28.75)	5.1384e-35^*^
t6	27.39 ± 4.99	27.73 ± 3.92	0.552
t7	29.62 ± 4.43	29.94 ± 4.03	0.565
t8	32.42 ± 4.31	32.27 ± 4.90	0.803
cc2	10.37 ± 2.27	9.24 ± 3.57	0.005^*^
s2	1.47 ± 1.77	2.37 ± 3.11	0.008^*^
cc3	9.89 (3.5, 11.25)	12.25 (10.25, 14.25)	6.541e-32^*^
s3	11.25 (7.76,15.5)	5.00 (3.5, 8.00)	1.5699e-35^*^

Note: t2, t3, t4, t5, t6, t7 and t8: The absolute times of division to 2, 3, 4, 5, 6, seven and 8 cells minus the tPNF, respectively. cc2: cleavage period time from 2- to 3-cell; s2: the duration of cell division from 3- to 4-cell; c3: time of the third cell cycle, cleavage from 3- to 5-cell; s3: the development of 5-cell embryos into 8-cell embryos.

Data are presented as the mean ± SD, or median (quartiles). The (*) values indicate the significant differences between the two groups (*p* < 0.05).

### Grading for sHLA-G expression

In this section, we verified the relationship between the level of sHLA-G secreted in D3 ECM by ELISA and embryo implantation results. The quantity of sHLA-G in each culture solution was interpolated from the corresponding optical density (OD, 450 nm) values. Outcomes of pregnancy facilitated the creation of a Receiver Operating Characteristic (ROC) curve, which displays an Area Under the Curve (AUC) of 0.71 (95% Confidence Interval [CI]: 0.644–0.776) (Refer to Supplementary Figure S1). The computed cut-off value is defined as “a = 2.903”, with a sensitivity and specificity of 61.2% and 78.9% respectively. Thus, levels equal to or exceeding 2.903 U/mL (the established cut-off value) were designated as sHLA-G positive, while concentrations less than 2.903 U/mL were classified as sHLA-G negative (see in Supplementary Material). Based on this classification, 136 embryos are sHLA-G positive and 102 are negative for sHLA-G.The implantation rates in the sHLA-G positive group were significantly elevated compared to the sHLA-G negative group (63.24% vs. 22.5%, *p* = 4.5338e-10).

### Embryo grades based on a classification model

In order to explore the potential influence factors, a multivariate logistic regression analysis was performed. And the results displayed that sHLA-G levels (OR 2.350; 95%CI 1.703–3.242; *p* < 0.001), t5(OR 0.854; 95%CI 0.763–0.955; *p =* 0.006), s3 (OR 1.247; 95% CI 1.122–1.387; *p* < 0.001) were independent risk factors for clinical pregnancy outcome. Therefore, t5 and s3 were subsequently chosen as candidate parameters to be included into the deselection model.

We divided t5 and s3 into four quartiles (Q1, Q2, Q3, Q4) and compared the implantation rate across each quartile interval with the objective of constructing a TLI hierarchical classification model. As showen in Table 3, the highest implantation rate was observed in the first quartile (Q1) of t5 and the last quartile (Q4) of S3, suggesting that late division events possess substantial predictive value for embryonic outcomes. The two quartiles exhibiting the highest frequency of embryo implantation were selected as the optimal range for both parameters.

**TABLE 3:** Quartile ranges of quantitative parameters and corresponding implantation rates after qualitative deselectionof t5 and s3 (*n* = 238).

Quartile	t5		s3	
	Range, h	Implantation rate (%)	Range, h	Implantation rate (%)
First	<20.19	79.67 (44/59)^c,d^	<4.25	16.67 (9/54)^c,d^
Second	20.19–24.38	56.67 (36/60)^c,d^	4.25–7.76	26.87 (18/67)^c,d^
Third	24.38–27.81	22.03 (16/59)^a,b^	7.76–11.75	65.45 (36/55)^a,b^
Fourth	≥27.81	25 (15/60)^a,b^	≥11.75	77.42 (48/62)^a,b^

Note: t5:time from pronuclear fading to 5-cell stage,s3: the development of 5-cell embryos into 8-cell embryos. ^a,b,c,d^ Same superscript indicates statistical significance compared to First, Second, Third quartile and Fourth quartile range, *p* < 0.05.

**FIGURE 1:**
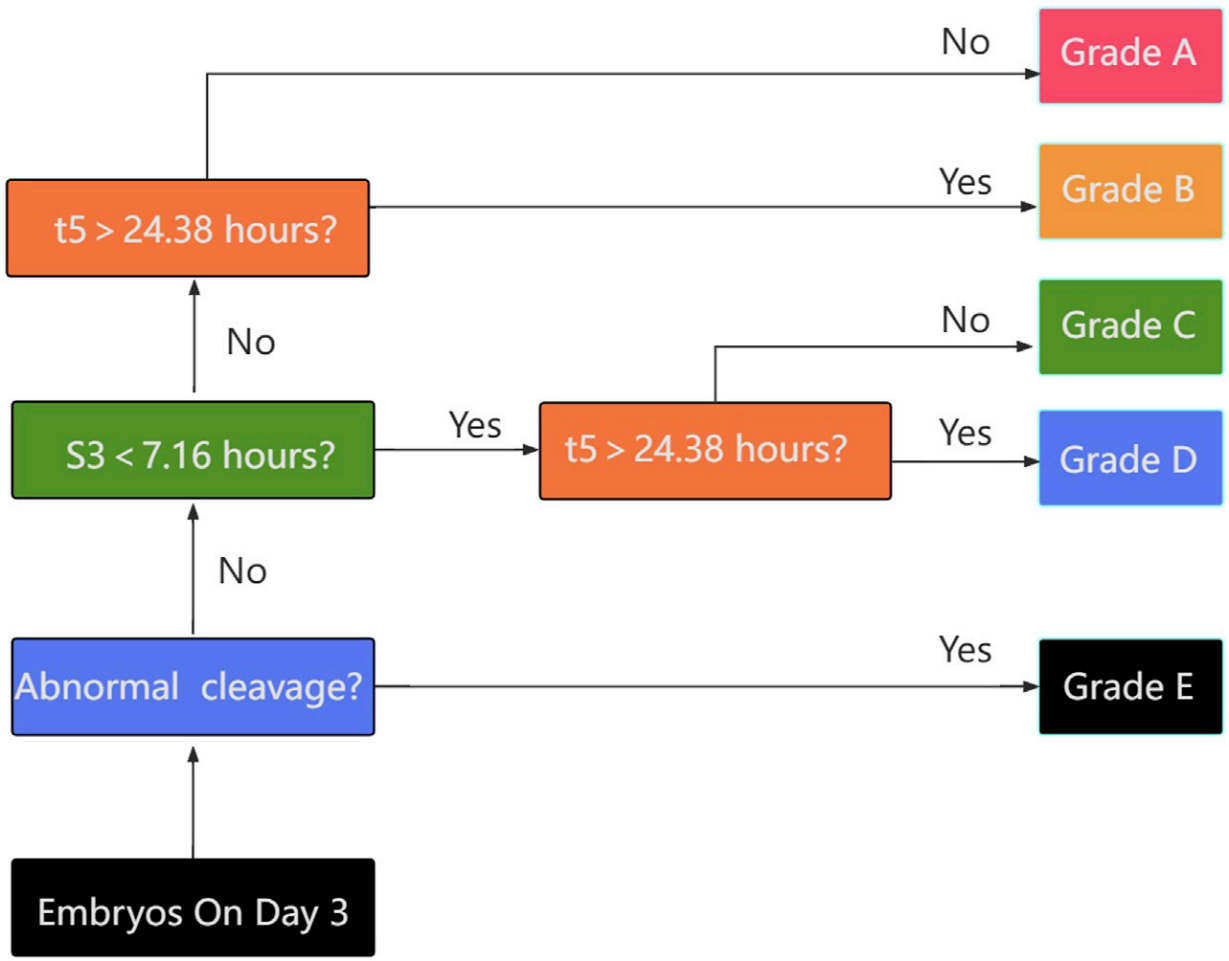
Flow chart for embryo classification based on: i) absence of exclusion criteria; ii) timing of the transition of 5-cells embryos to 8-cells embryos (s3); iii) timing of the cell division to 5 cells (t5). The classification generates 5 categories of embryos with decreasing expected implantation potential (upper to lower).

From the results obtained, a hierarchical TLI classification model was suggested in this study to identify embryos with high implantation. Embryos were categorized into five grades from A to E as described in Figure 1, in details, those embryos with irregular division pattern, such as a direct or rapid cleavage from 1 to 3 cells (i.e., CC2<5 h), or DUC or ICD or RC, were defined as category E. The subsequent levels in the model adhered to a hierarchy based on the binary timing variables t5 and s3. An embryo was classified as either A or B if the value of s3 was within the optimal range (≧7.76 h). Conversely, if the value of s3 was outside this range, the embryo was classified as either C or D. Depending on the value of s3, an embryo was classified as either A or C if the value of t5 was within the optimal range (<24.38 h), and as either B or D if the value of t5 was outside this range. As per the Time-lapse Imaging (TLI) classification model, a downward trend in the rate of embryo implantation was documented with the decline in the grade: Grade A demonstrated an implantation rate of 86.42% (70/81), Grade B was at 58.33% (14/24), Grade C was 34.78% (8/23), Grade D sank to 16.47% (14/85), and Grade E bottomed out at 12.0% (3/25) (intergroups *p* = 3.4434e-20) as represented in Table 4.

**TABLE 4:** Implantation rate and sHLA-G positive embryo rate of diverse grades according to the TLI classification model.

	A (*n* = 81)	B (*n* = 24)	C (*n* = 23)	D (*n* = 85)	E (*n* = 25)	*p*-value
Implantation rate (%)	86.42 (70/81)	58.33 (14/24)	34.78 (8/23)	16.47 (14/85)	12.00 (3/25)	3.4434e-20^*^
sHLA-G positive embryo rate (%)	76.54 (62/81)	58.33 (14/24)	52.17 (12/23)	47.06 (40/85)	32.00 (8/25)	0.0001^*^

Note: HLA-G:Soluble human leukocyte Antigen G; The (*) values indicate the significant differences (*p* < 0.05).

### Validation of the TLI classification model with KID

**FIGURE 2:**
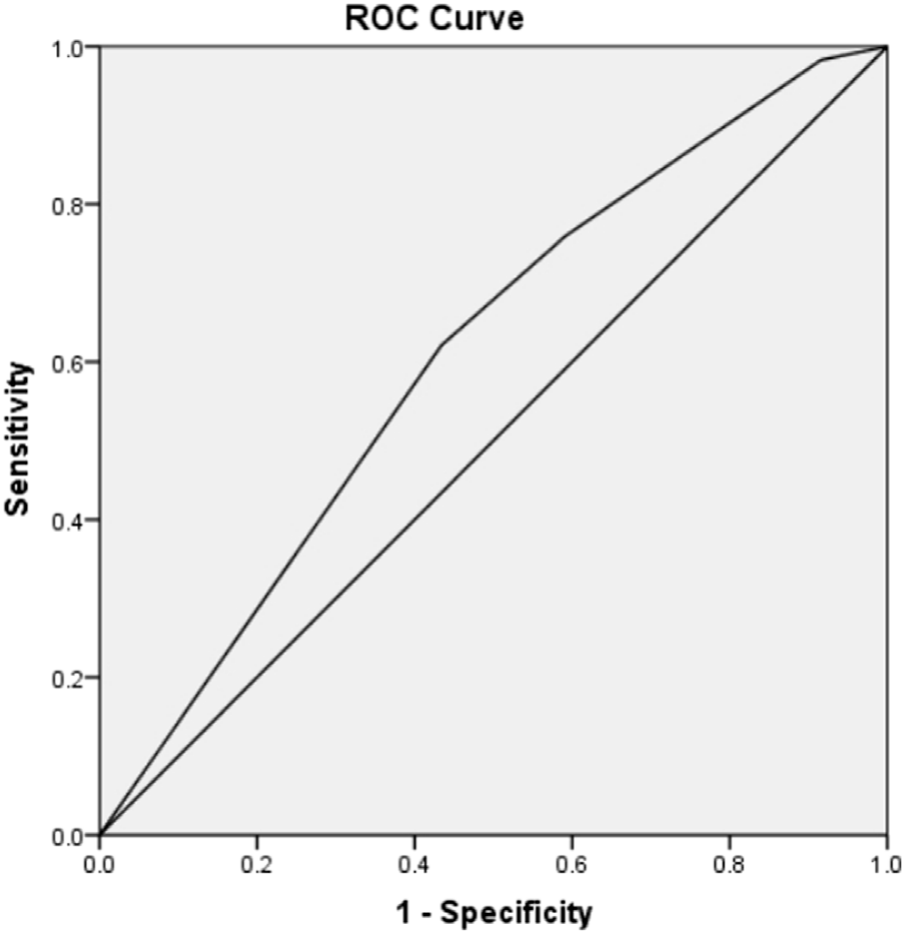
ROC curve to estimate the predictive value of TLI classification model which validated on an independent data set composed of 152 embryos with known implantation data (KID). (AUC = 0.609; 95% CI 0.516–0.703).

After the conclusion of the implantation potential of embryos is associated with a tightly regulated sequence of cellular events, the model presented here was validated on an independent data set composed of 152 embryos with known implantation data (KID embryos). Consistent with the above results, it was observed that as the grade decreased, the rate of embryo implantation also progressively declined: Grade A was 50% (22/44), Grade B was 43.75% (14/32), Grade C was 38.1% (8/21), Grade D was 28.89% (13/45), and Grade E was 20.00% (2/10). At the same time, the live birth rate also presents a consistent conclusion with the implantation rate, Grade A was40.91% (18/44), Grade B was 31.25% (10/32), Grade C was 28.57% (6/21), Grade D was 24.44% (11/45), Grade E still had no live births (0/25). Receiver Operating Characteristic (ROC) curve was utilized to evaluate the predictive validity which gives a AUC of 0.609 (95% CI 0.516–0.703) (Figure 2). The results verified the predictive power of the model.

### Comparison of sHLA-G expression among TLI classification categories

Here we compared the expression of sHLA-G in different levels (A-E) of the timelapse parameter model. As showed in Table 4, we observed a gradual decrease in the percentage of embryos with positive sHLA-G expression from A to E (76.54%, 58.33%, 52.17%, 47.06%, 32.00%, *p* = 0.0001).

Next, we further compared the implantation rates of embryos with positive sHLA-G expression *versus* embryos with negative sHLA-G expression in each level (A-E) of the timelapse parameter model. As showen in Table 5, the implantation rates of embryos with positive sHLA-G expression gradually decreased with the change in timelapse parameter level (A-E), and these differences were also found to be statistically significant. Additionally, even within the same timelapse parameter level A and B, embryos with positive sHLA-G expression had statistically significant higher implantation rates compared to embryos with negative sHLA-G expression.

**TABLE 5:** Comparison of implantation rate of sHLA-G positive or negative embryos in each grade.

Embryo grade	Positive/negative of sHLA-G	No. of transferred embryos(n)	No. of implantation embryos(n)	Implantation rate (%)	*p*-value
A	Positive	62	58	93.55	0.003^*^
	Negative	19	12	63.16	
B	Positive	14	11	78.57	0.035^*^
	Negative	10	3	30.00	
C	Positive	12	6	50.00	0.193
	Negative	11	2	18.18	
D	Positive	40	9	22.50	0.157
	Negative	45	5	11.11	
E	Positive	8	2	25.00	0.231
	Negative	17	1	5.88	

Note: HLA-G:Soluble human leukocyte Antigen G.The (*) values indicate the significant differences (*p* < 0.05).

**FIGURE 3:**
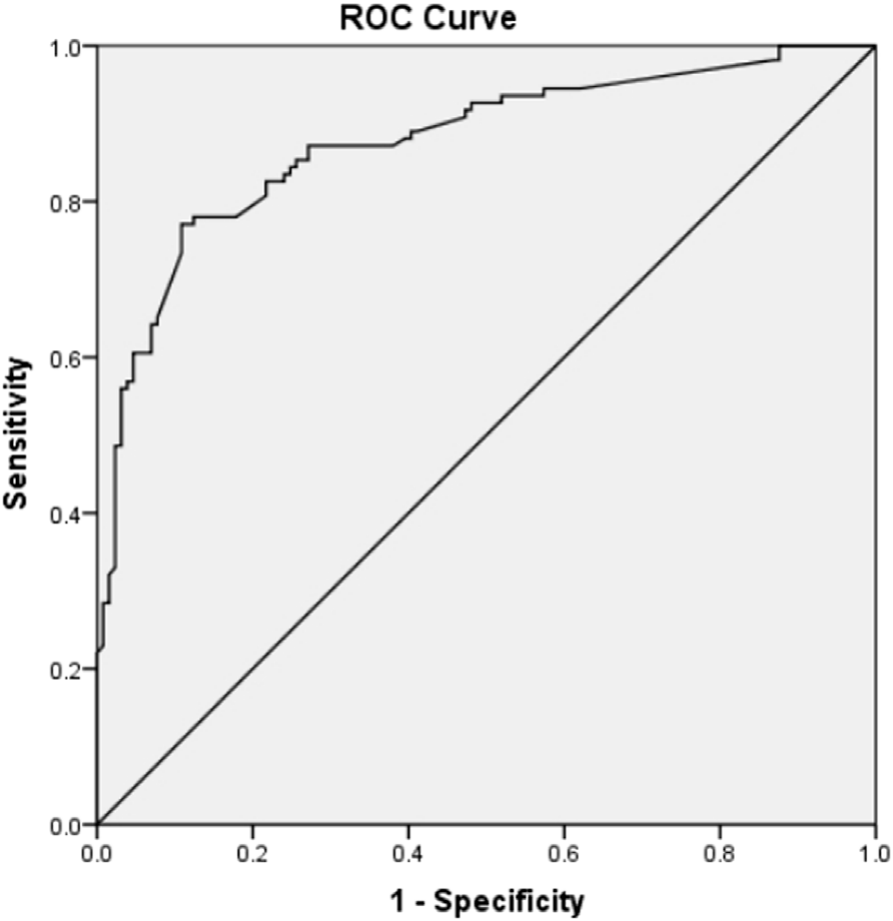
ROC curve to estimate the predictive value of sHLA-G in D3 medium of embryos combined with TLI classification model for embryo implantation. (AUC = 0.876; 95% CI 0.830–0.922).

sHLA-G may be utilized as a secondary parameter in the event of a requirement to choose between embryos exhibiting identical morphological or kinetic quality. ROC curve was employed to evaluate the predictive validity of HLA-G combined with TLI Classification Mode (Figure 3), which gives a AUC of 0.876 (95% CI: 0.830–0.922).

## Discussion

Embryo selection is a pivotal factor in IVF success and continues to be a challenge. Enhancements in predictive capabilities could aid in optimizing implantation success rates and other clinical outcomes, thereby potentially reducing the financial and emotional stress imposed on patients. Previous studies have demonstrated significant interobserver variability amongst embryologists when assessing embryo quality and the ending of embryos may differ. In recent years, many TLI systems have been widely used. It can provide embryologists with abundant information about the key time points during the development process of embryo growth for embryo selection (Meseguer et al., 2011). Several morphokinetic algorithms have been developed in the attempt to make the prediction of clinical or laboratory outcomes more accurate (Petersen et al., 2016). Implantation and live birth are certainly the clinical endpoint more investigated. Algorithms were also developed to predict the risk of aneuploidy, as assessed by preimplantation genetic testing for aneuploidy (PGT-A) performed at the blastocyst stage on trophectoderm cells, although it still can’t replace PGT-A (Balakier et al., 2016; Minasi et al., 2016; Kimelman et al., 2019; Lee et al., 2019). Embryologists demonstrate a minimum level of fair consensus when tasked with evaluating the probability of blastocyst implantation utilizing TLI (Fordham et al., 2022). However, it is noteworthy that high heterogeneity in published TLI classification algorithms together (Storr et al., 2018). Algorithms/models may be successful in the units where they were developed, but not always possible to replicate results in other clinical settings. That why today we still lack of consensus surrounding TLI.

In this study, we aimed to build a TLI model by retrospective analysis implantation outcomes of FET in our own center to enhance the value of information derived from embryo morphokinetics. In our practice, good quality embryos are usually selected first in the fresh embryo transfer cycles or the FET with a previous fresh transfer. Meanwhile, there may be a difference in embryo quality between those different cycles, so we chose freeze-all cycles.

In this study, tPNF was used as the starting point, and eleven time parameters including t2 to t8, cc, s2, cc3, and s3 were compared between embryos that implanted and those that did not. Binary logistic regression analysis indicated that two kinetic parameters, S3 and t5, affected the embryo implantation rate, and their optimal intervals had predictive significance for embryo implantation. Numerous studies have demonstrated that early parameters of embryonic development, such as the time of pronuclear formation and disappearance, the first division time, and the time from 3-cell to 4-cell, are crucial throughout the embryonic development process. These parameters are related to blastocyst formation or embryo implantation, and embryos that divide relatively early have a higher chance of developing into blastocysts. A retrospective report in a single IVF center indicated that embryos competent to provide a live birth display overall faster early developmental kinetics including tPNf, t2 compared with embryos that do not achieve a live birth after transfer (Dal Canto et al., 2021). Previous research has demonstrated that direct division and reverse division exert an influence on embryonic development, thereby resulting in a diminished capacity for embryo implantation, although embryologists disagree on whether abnormally divided embryos can be born alive (Aguilar et al., 2016; Almagor et al., 2016; Fan et al., 2016). Embryos exhibiting abnormal cleavage indicate a possibility of implantation but minimal live birth. In a retrospective cohort study included 1562 consecutive autologous *in vitro* fertilization cycles, abnormal cleavage up to Day 3 is associated with reduced full blastulation rates but does not impact live birth rates and neonatal outcomes once full blastulation has been achieved (Lee et al., 2024). In preimplantation genetic testing cycles, blastocysts demonstrating either direct or reverse cleavage should be biopsied, provided they meet morphological eligibility criteria. It is essential to note, however, euploid blastocysts displaying abnormal cleavage patterns exhibit approximately half the live birth rates (LBR) compared to their counterparts with normal cleavage. Consequently, transfer priority should be lowered for blastocysts showing abnormal cleavage patterns (Ozbek et al., 2021). Thus, despite there being only 25 cases, we still defined embryos with an irregular division pattern and classified them as E. The implantation rate, clinical pregnancy and live birth rate in level E embryos showed 30%, 40%, and 0%, respectively. For Day-3 embryos with abnormal cleavage, it is recommended to undergo blastocyst culturing, rather than priority implantation. This aids in the selection of high-quality embryos, consequently enhancing the rate of successful births. The underlying mechanisms and successful implantation processes of abnormally cleaved embryos, and whether they can achieve live births as normally developing embryos do, still require in-depth exploration. This study did not find a significant relationship between early embryonic development parameters and implantation rates. This may be due to tPNF was used as the starting point, which could potentially lead to discrepancies in results when compared with other researchers. Multicenter experimental study demonstrated that embryos with a cc2 less than 5 h had a significantly lower implantation rate than embryos with a normal cleavage pattern (Rubio et al., 2012). Desai et al. reported that RC significantly compromised embryo development, culminating in poor implantation potential (Desai et al., 2014). We found that t5 (the time cleavage from tPNf to the 5-cell stage) and s3 (the transition of 5-cell embryos to 8-cell embryos) were independent factors for pregnancy outcome by a logistic regression analysis. The result were similar to Meseguer and Motato, who also found that s3 was one of the most predictive parameters for blastocyst formation (Meseguer et al., 2011; Motato et al., 2016). Meseguer’s model incorporated three variables (t5, s2 and cc2) to subdivide embryos into 10 categories (Meseguer et al., 2011). The optimal intervals of S3 and t5 were combined with abnormal cleavage events to establish a preliminary embryo TLI classification model in our center. This model, in which embryos were categorized into five grades from A to E, was validated by kid and the results showed that the embryo implantation rate gradually decreased with the decline in embryo grade, with an AUC of 0.609.

HLA-G molecules play a key role in the establishment and maintenance of immune tolerance (Carosella et al., 2008). Several reports have described an association between the presence of HLA-G in human embryo ECM and implantation success (Fuzzi et al., 2002; Noci et al., 2005; Shaikly et al., 2010; Díaz et al., 2019; Radwan et al., 2022). Taking into account the rates of achieved pregnancies and live births, sHLA-G secreted by the embryo may be a factor supporting implantation. In the research conducted by Desai and colleagues, it was demonstrated that when at least one embryo chosen for transfer tested positive for sHLA-G, the rate of successful pregnancies achieved was 64%, with an implantation rate per transferred embryo of 38%. Conversely, in patients who received solely sHLA-G negative embryos, there was a marked decrease in both the pregnancy rate, which stood at 36%, and the implantation rate, which was recorded at 19% (Desai et al., 2006). Radwan et al. investigated the concentration of sHLA-G in media from 344 single cultured embryos following IVF/ICSI, and clearly showed that the concentration of sHLA-G secreted by embryos depends on multiple parameters, including: the day of measurement, the amount of collected material, the procedure of ovarian stimulation (Radwan et al., 2022). Higher levels of sHLA-G are potentially associated with pregnancy success. Nevertheless, these findings are not universally agreed upon. A multicenter blinded study, which included 1405 ECM from 355 patients across three ART centers, demonstrated substantial variations in the HLA-G content in ECM between different ART centers. This underscores the influence of various technical parameters that can differ from one center to another (Tabiasco et al., 2009). In this part of the analysis, we examined the level of sHLA-G secreted by the embryos and its potential influence on reproductive success. Our results are concordant with the result that embryos in pregnancy and/or live birth secrete more sHLA-G compared to those whose transfer ends without pregnancy. Other published studies including a multicenter study are also in line with our research (Sher et al., 2004; Sher et al., 2005; Rebmann et al., 2010; Vani et al., 2021). Besides, we compared the expression of sHLA-G in different levels of the timelapse parameter model. It was observed that embryos exhibiting positive sHLA-G expression at the same timelapse parameter level had significantly elevated implantation rates, which was consistent with Rebmann et al. that HLA-G can be considered a second parameter if a choice must be made between embryos of the same morphological quality (Rebmann et al., 2010).

In conclusion the integration of sHLA-G level in D3 ECM with the above TLI classification predictive model may serve as a novel non-invasive approach for quality embryo selection. However, this study is also restricted by its retrospective nature and sample size. Prospective randomized controlled trials should be used in future studies to validate the value of this novel non-invasive predictive model.

## Data Availability

The raw data supporting the conclusion of this article will be made available by the authors, without undue reservation.
