# Supplementary material for: The synergy of morphokinetic parameters and sHLA-G in cleavage embryo enhancing implantation rates
Source: Front Cell Dev Biol. 2024 Jul 16;12:1417375. doi: 10.3389/fcell.2024.1417375 (PMC11286472; doi:10.3389/fcell.2024.1417375)
Supplement: Supplementary file 1 [file DataSheet1.docx]

**Supplemental information**

**Supplemental Figure 1**


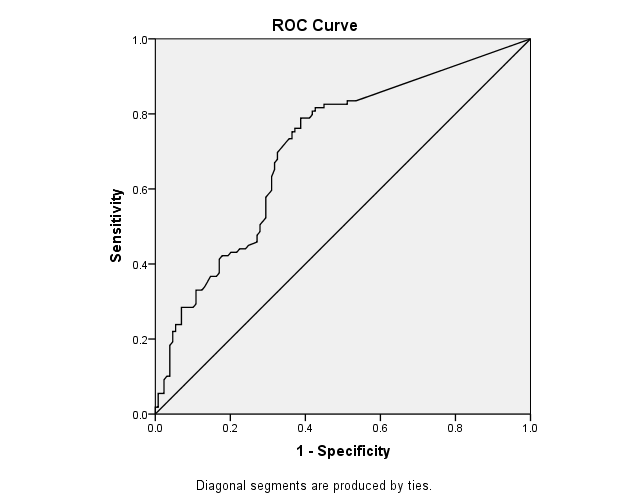


Utilizing the dilution multiples of the sHLA-G standard, a linear regression formula linking sHLA-G concentration and optical density (OD, 450nm) was created. The quantity of sHLA-G in each culture solution was interpolated from the corresponding OD values. Outcomes of pregnancy facilitated the creation of a Receiver Operating Characteristic (ROC) curve, which displays an Area Under the Curve (AUC) of 0.71 (95% Confidence Interval [CI]: 0.644-0.776) (Refer to Figure 1). The computed cut-off value is defined as 'a = 2.903', with a sensitivity and specificity of 61.2% and 78.9% respectively. Thus, values that are greater or equal to 'a' are classified as sHLA-G positive, whereas values lesser than 'a' are deemed negative. Based on this classification, 136 embryos are sHLA-G positive and 102 are negative for sHLA-G.

**Supplemental Table 1**

Multiple logistic regression analysis of the association between the confounding variables relating to the implantation of embryos.

|  | P-value | Odds ratio (95% CI) |
| --- | --- | --- |
| No.of antral follicle | 1.022 | 1.022(0.902-1.156) |
| No. of retrieved oocytes | 1.135 | 1.135(0.919-1.1403) |
| No. of MII oocytes | 0.945 | 0.945(0.736-1.215) |
| sHLA-G levels | <0.001^*^ | 2.350(1.703-3.242) |
| t3 | 0.192 | 1.375(0.852-2.220) |
| t5 | 0.006^*^ | 0.854(0.763-0.955) |
| cc2 | 0.767 | 0.932(0.585-1.485) |
| s2 | 0.353 | 1.110(0.891-1.383) |
| s3 | <0.001^*^ | 1.247(1.122-1.387) |

*Note: HLA-G:Soluble human leukocyte Antigen G ;CI: Confidence Interval.; t3, t5：The absolute times of division to 3, and 5 cells minus the tPNF, respectively. cc2：the length of the 2-cell period, cleavage from 2- to 3-cell, s2： the duration of cell division from 3- to 4-cell, s3：the development of 5-cell embryos into 8-cell embryos. The (*) values indicate the significant differences (p < 0.05).*

**Supplemental Table2**

Validation on an independent data set composed of 152 embryos with KID.

| Embryo Grades | Embryos transferred (n) | Implantation rate(%) | Live birth rate(%) |
| --- | --- | --- | --- |
| A | 44 | 50.00(22/44) | 40.91(18/44) |
| B | 32 | 43.75(14/32) | 31.25(10/32) |
| C | 21 | 38.10(8/21) | 28.57(6/21) |
| D | 45 | 28.89(13/45) | 24.44(11/45) |
| E | 10 | 20.00(2/10) | 0(0/10) |
|  |  |  |  |
